# Integrated transcriptomics and metabolomics provide insights into the biosynthesis of militarine in the cell suspension culture system of *Bletilla striata*

**DOI:** 10.1007/s44307-024-00032-w

**Published:** 2024-07-16

**Authors:** Qingqing Li, Mengwei Xu, Fengju Wu, Ziyi Guo, Ning Yang, Lin Li, Weie Wen, Delin Xu

**Affiliations:** 1 Department of Medical Instrumental Analysis, Zunyi Medical University, Zunyi, 563099 Guizhou China; 2 Department of Cell Biology, Zunyi Medical University, Zunyi, 563099 Guizhou China

**Keywords:** Militarine, Elicitor, Suspended calluses, Multiomics, Molecular mechanism

## Abstract

Militarine is a monomer molecule with abundant and distinctive biological properties, also the lead member of secondary metabolites in *Bletilla striata*, while its biosynthesis mechanism is still unknown. To improve the production efficiency of militarine, sodium acetate and salicylic acid (SA) were introduced as elicitors into the suspension-cultured callus of *B. striata*. Subsequently, samples were taken from callus at different culturing stages to investigate the synthesis mechanisms of militarine in *B. striata* through integrated metabolomics and transcriptomics. Metabolomics analysis revealed that acetate ions promoted militarine synthesis, while SA had an inhibitory effect. Additionally, regulators such as ferulic acid, 2-hydroxy-3-phenylpropionic acid, and cis-beta-D-Glucosyl-2-hydroxycinnamate were identified as influencing militarine synthesis. Transcriptomics analysis indicated that the expression levels of genes involved in phenylalanine metabolism, phenylpropanoid biosynthesis, and tyrosine metabolism were correlated with militarine content. This study sheds light on the regulatory mechanism of militarine biosynthesis in plants. The results suggested that acetate ions and SA impact militarine synthesis through specific metabolic pathways and gene expression changes. This knowledge serves as a foundation for future research on militarine biosynthesis and its industrial production.

**Supplementary Information:**

The online version contains supplementary material available at 10.1007/s44307-024-00032-w.

## Highlights

1. Elicitation modulates militarine biosynthesis in *Bletilla striata*.

2. Omics reveals key metabolites and genes involved in militarine synthesis.

3. Differential gene expression correlates with militarine content.

4. Insights for enhanced militarine production.

**Supplementary Information:**

The online version contains supplementary material available at 10.1007/s44307-024-00032-w.

## Introduction

*Bletilla striata* (Thunb.) Rchb. f., a perennial herb within the Orchidaceae family, is distinguished by its diverse pharmacologically active constituents (Xu et al. 2019; Pan et al. 2018). Militarine, a glycosidic compound, is one of the main active ingredients in *B. striata* that contributes to its effective use (Jia et al. 2024)*.* Research had shown that militarine possesses significant neuroprotective effects (Fisher et al. 2014) and had the potential to improve cognitive impairment in the brain (Zhang et al. 2023a, b) as well as inhibit inflammation-induced respiratory system damage (Chen et al. 2016). Militarine had been identified as possessing potent hemostatic properties. At a dose one-tenth that of Yunnan Baiyao, it achieves a hemostatic effect comparable to the reference standard (Guo et al. 2016). Further research suggested militarine's promise as a reversal agent for heparin-induced anticoagulation in clinical applications (Yan et al. 2014). As a result, it had attracted considerable attention from researchers and had tremendous commercial prospects. Traditionally, there had been three approaches to acquire militarine: isolating and extracting it from the dried tuberous roots of *B. striata* (Li et al. 2023a), synthesising it chemically or enzymatically (Jaiswal et al., 2022), and producing it through biosynthesis (Scheler et al. 2016). In vitro biosynthesis refers to the use of enzymatic reactions to manufacture natural chemicals, by harnessing the cell's inherent metabolic pathways to control the synthesis of specific substances (Li et al. 2023c). This approach was regarded as secure, effective, and shows great potential for future advancement.

The aim of this research was to elucidate the impact of salicylic acid (SA) and sodium acetate (NaAc) on the production of militarine, by modulating essential biosynthetic pathways, employing transcriptomics and metabolomics analyses. This project aimed to establish the foundation for understanding the genetic mechanisms that control the production of militarine in *B. striata*. Elicitors were known to stimulate the production of target products in plant cells (Chen 2023; Liu et al. 2021). Applying external elicitors to plants had been discovered to increase the synthesis of secondary metabolites. According to Rauch et al. (2019), SA had the capacity to increase the expression of important genes involved in glycoside production. This entity engages in many signal transduction mechanisms (Hansen et al. 2012), governs plant reactions during biotic or abiotic stress circumstances, and enhances the buildup of secondary metabolites in plants (Yu et al. 2019). NaAc was involved in the metabolic pathway, as well as its subsidiary process of phenylpropane, which was intricately linked to amino acid metabolism. Supplementing the culture medium with NaAc can augment the production and accumulation of secondary metabolites, such as flavonoids and glycosides (Zhang et al. 2020). At now, there was a lack of reports on the impact of NaAc and SA on suspension cultured calluses of *B. striata*.

The research established suspension cultures of *B. striata* calluses and treated them with SA and NaAc. Over time, researchers monitored the cultures to capture the dynamics of gene expression and metabolite production, focusing on the crucial stages of militarine synthesis. Our findings revealed a significant upregulation of genes involved in the glycoside biosynthesis pathway upon elicitor treatment.

## Materials and methods

### Materials

The experimental material comprised suspension-cultured calluses of *B. striata*. Mature capsules were induced in the appropriate medium for 30 days, after which, soft, yellow, tender tissue calluses were selected for transfer to a subculture medium. The medium was refreshed every 15 days. Commencing with the second subculture (recorded as 0 days post-inoculation, dpi), calluses were treated with 150 μmol/L sodium acetate or 15 μmol/L SA independently. Different growth stages (3, 18, 21, 36 dpi), of suspension calluses were sampled randomly, adhering to a randomized design with three replicates per stage. Total RNA was isolated, quality-verified, immediately freeze-dried in liquid nitrogen, and preserved at -80 °C for subsequent analyses (Liu et al. 2020).

### Culture of suspended calluses

Select fully filled and undamaged mature capsules and disinfect them. The contents were subjected to a gentle agitation within a 15 ml test tube. Subsequently, a 75% ethanol solution was introduced for the purpose of disinfection, with a duration of 60 s. The specimens were thoroughly washed on three occasions using sterile water. Subsequently, a solution of mercuric chloride with a concentration of 0.1% (including 1 mg/L of Tween-80) was introduced for disinfection purposes, and left for a duration of 30 s. The specimens were thoroughly washed on three occasions using sterile water. Eventually, the sterile water was disposed of and placed to the side. The seeds were uniformly distributed in the induction medium, which consisted of MS supplemented with 1 mg/L 6-BA, 2 mg/L 2,4-D, 0.5 mg/L NAA, and 30 g/L sucrose. Following a 30-day period of induction culture, a subculture was conducted. The yellow callus tissue was chosen and introduced into the proliferation medium, which consisted of 1/2 MS, 1 mg/L 6-BA, 3 mg/L 2,4-D, 0.5 mg/L NAA, and 30 g/L sucrose. Subculturing was conducted at a frequency of every 15 days. The culture was maintained at a temperature of (25 ± 1) °C, in the absence of light, with a shaking speed of 120 revolutions per minute (Liu et al. 2022).

Commencing from the second subculture (0 dpi), varying concentrations of elicitors (as listed in Table 1) were introduced into the culture media for successive cultivation. The initial amount of inoculum was 1 gramme, and the suspension calluses were collected after the fourth passage, which took 45 days post-inoculation (dpi). The experiments were replicated three times. The dried calluses were utilised as a source material for the isolation and analysis of secondary metabolites, such as militarine. The most effective concentration of elicitors was chosen based on the content of militarine. The cultivation was conducted at the ideal concentration for a duration of 45 days. Samples were collected every 3 days, with each sampling event repeated three times.

**Table 1:** Suspension callus culture scheme of *Bletilla striata*

Group	**Culture medium + Plant growth regulator (mg/L)**				**Elicitor (μmol/L)**		
	**Basal medium**	**6-BA**	**2,4-D**	**NAA**	**NaAc**	**NaCl**	**SA**
1	1/2MS + 30 g/L sucrose	1	3	0.5	0	-	-
2	1/2MS + 30 g/L sucrose	1	3	0.5	50	-	-
3	1/2MS + 30 g/L sucrose	1	3	0.5	100	-	-
4	1/2MS + 30 g/L sucrose	1	3	0.5	150	-	-
5	1/2MS + 30 g/L sucrose	1	3	0.5	200	-	-
6	1/2MS + 30 g/L sucrose	1	3	0.5	-	0	-
7	1/2MS + 30 g/L sucrose	1	3	0.5	-	50	-
8	1/2MS + 30 g/L sucrose	1	3	0.5	-	100	-
9	1/2MS + 30 g/L sucrose	1	3	0.5	-	150	-
10	1/2MS + 30 g/L sucrose	1	3	0.5	-	200	-
11	1/2MS + 30 g/L sucrose	1	3	0.5	-	-	0
12	1/2MS + 30 g/L sucrose	1	3	0.5	-	-	5
13	1/2MS + 30 g/L sucrose	1	3	0.5	-	-	10
14	1/2MS + 30 g/L sucrose	1	3	0.5	-	-	15
15	1/2MS + 30 g/L sucrose	1	3	0.5	-	-	20
16	1/2MS + 30 g/L sucrose	1	3	0.5	-	-	25
17	1/2MS + 30 g/L sucrose	1	3	0.5	-	-	50
18	1/2MS + 30 g/L sucrose	1	3	0.5	-	-	75
19	1/2MS + 30 g/L sucrose	1	3	0.5	-	-	100
20	1/2MS + 30 g/L sucrose	1	3	0.5	-	-	150
21	1/2MS + 30 g/L sucrose	1	3	0.5	-	-	200

### Determination of militarine content

*B. striata* suspension calluses were taken, dried, ground in a mortar, and passed through a 65-mesh sieve. A precise amount of 0.20 g was weighed and placed in a round-bottom flask. 100 ml of 70% methanol–water was added and refluxed for 2 h. The extract was filtered using filter paper to obtain the extract. The extract was then evaporated to dryness using a rotary evaporator, dissolved in an appropriate amount of 70% methanol (analytical grade), transferred to a 5 ml volumetric flask, and made up to volume. After thorough mixing, the solution was filtered through a 0.45 µm filter membrane to obtain the test solution. The sample was injected for analysis and detection of secondary metabolite content (Liu et al. 2022).

Use Waters 2695–2489 HPLC instrument (high-performance liquid chromatography) for sample analysis. The chromatographic conditions used in the study were as follows: the column used was University C18 with dimensions of 250 mm × 4.6 mm and a particle size of 5 µm. The flow rate of the mobile phase was set at 0.8 mL/min. The mobile phase consisted of 100% acetonitrile (A) and 0.1% phosphoric acid (B). The column temperature was maintained at 25 ℃. The detection wavelength used was 223 nm. A sample injection volume of 10 μL was used (Liu et al. 2022). The table S1 displayed the variation in the volume fraction of the solvent throughout the gradient elution operation (Detector:2998).

### Transcriptome sequencing and bioinformatics analysis

Transcriptome sequencing was entrusted to Hangzhou Lianchuan Biotechnology Co., Ltd. After verifying the quantity, purity, and integrity of the total RNA, the mRNA containing PolyA (polyadenylate) was specifically captured using oligo (dT) magnetic beads (Dynabeads Oligo (dT)), followed by the use of a magnesium ion disruption kit (NEBNext). Magnisium RNA Fragmentation Module was subjected to fragmentation at 94 ℃ for 5–7 min. Fragments were processed through reverse transcriptase (Invitrogen SuperScript ™ The function of II Reverse Transcriptase is to synthesize cDNA. Then, double-stranded synthesis was performed using DNA polymerase I (*E. coli*) and RNase H to convert the complex double-stranded DNA and RNA into DNA double-strands. dUTP solution (Thermo Fisher) was also incorporated into the double-stranded DNA to blunt-end the double-stranded DNA. An A base was added to each end, and fragment size selection was performed using magnetic bead screening and purification. The second strand was digested with UDG enzyme, followed by PCR amplification (95 °C pre-denaturation for 3 min; 98 °C denaturation for 15 s, 8 cycles; 60 °C annealing for 15 s; 72 °C extension for 30 s; 72 °C final extension for 5 min) to generate a library with fragments of approximately 300 bp ± 50 bp in size. Lastly, Illumina NovaSeq™ 6000 (LC Biotechnology Co., Ltd., China) was used for paired-end sequencing according to standard protocols, with a sequencing mode of PE150.

Offline data were filtered (in Fastq format) using Fastp (Chen et al. 2018), filtered data were de novo assembled using Trinity2.4.0 (Grabherr. et al., 2011), and assembled transcripts were clustered based on sequence similarity. The longest fragment of these similar transcripts was then selected as Unigene. All assembled Unigenes were compared and annotated with Nr library, Gene Ontology (GO), SwissProt, Kyoto Gene and Genome Encyclopedia (KEGG), and eggNOG using DIAMOND (Buchfink et al. 2015) with annotation comparison thresholds < 1 × 10^–5^. Using Salmon (Patro et al. 2017) to quantify Unigenes using TPM (Mortazavi et al. 2008), and using R-packet edge R for differential analysis (Robinson et al. 2010), Unigenes with multiple differences greater than 2 or less than 0.5 and *p* < 0.05 were all differential genes.

### Metabolome sequencing and analysis

The samples were defrosted on ice and the metabolites were extracted from 20 µL of each sample using 120 µL of a pre-cooled buffer containing 50% methanol. The mixture of metabolites was agitated vigorously for a duration of 1 min, followed by an incubation period of 10 min at room temperature. The mixture was then stored at a temperature of -20 ℃ for the whole duration of the night. The combination underwent centrifugation at a force of 4000 times the acceleration due to gravity for a duration of 20 min, resulting in the transfer of the liquid portion (supernatant) to a 96-well plate. After combining 10 µL of each extraction mixture, they were utilised as pooled quality control (QC) samples. The samples underwent analysis utilising a Triple TOF 5600Plus high resolution tandem mass spectrometer (SCIEX, Wallington, UK). An Ultra Performance Liquid Chromatography (UPLC) equipment from SCIEX, UK was used to achieve the chromatographic separation. The precision of quality control was calibrated at regular intervals of every 20 samples for the whole collection period. Furthermore, a single quality control (QC) sample was analysed for every 10 samples in order to evaluate the stability of the LC–MS.

The data sets underwent normalisation before analysis. The data for all samples was normalised using the probability quotient normalisation procedure. Subsequently, the QC samples were employed for QC robust spline batch calibration. The *P*-values were evaluated using a T-test, and then adjusted for multiple testing using the False Discovery Rate (FDR) method proposed by Benjamin Hochberg. This modification was performed to account for the selection of distinct metabolites. The software MetaX was employed to monitor the PLS-DA variables, which were subjected to discriminant analysis statistical techniques in order to pinpoint more precise distinctions between the groups. A threshold of 1.0 was employed to select significant functions for VIP.

### DEGs and DAMs screening

Make use of transcriptome and metabolome sequencing databases to identify genes that were expressed differently (DEGs) and metabolitesthat were present in varying quantities (DAMs). Genes that showed differential expression were identified by applying criteria such as *p* < 0.05, fold change (FC) greater than 2, or less than -2. Univariate analysis was performed using t-test statistical tests. The q value was calculated using the Benjamini/Hochberg method for false discovery rate (FDR) correction. The selection process involved integrating multivariate statistical analysis of VIP (predicted important variable) values derived using partial least squares regression (PLS-DA). Differential metabolites meeting the criteria of ratios ≥ 2 or < 1/2, *p* ≤ 0.05, and VIP ≥ 1 were chosen. Utilising KEGG pathway annotation to identify common elements and evaluate significant genes and metabolites using a significance threshold of *p* < 0.05 and a correlation coefficient threshold of *r* > 0.8. Retrieve metabolic pathway maps from the KEGG database and utilise the R programming language to generate heat maps.

### qPCR validation

The initial cDNA strand was produced using reverse transcription utilising the TIANGEN kit. The template for this process consisted of 1 μg of total RNA extracted from the examined samples at 3 dpi, 18 dpi, 21 dpi, and 36 dpi. Conduct a qPCR experiment using the AGS 4800 real-time quantitative PCR apparatus. The qPCR reaction apparatus has a total capacity of 10 μL. The components used in the experiment were 1 μL of cDNA template with a concentration range of 1–10 ng/μL, 5 μL of SYBR Green qPCR Master Mix, 0.2 μL of primers (each primer with a concentration of 10 μmol/L), and 3.6 μL of ddH_2_O. Choose nine genes at random for validation and utilise Primer Blast on the NCBI website to create targeted primers for the qPCR reaction of the genes (Table S2). The reaction was conducted using the following parameters: initial denaturation at a temperature of 95 °C for a duration of 30 s, followed by 40 cycles of amplification consisting of denaturation at 95 °C for 10 s, annealing at 60 °C for 30 s, and extension at 72 °C for 30 s. Finally, the reaction was concluded with an extension step at 95 °C for 15 s. Apply the 2^− ΔΔ Ct^ technique to determine the relative expression of genes, using three biological and technical replicates for each group (Liu et al. 2022).

## Results

### Determination of militarine content in suspension calluses

**Fig. 1:**
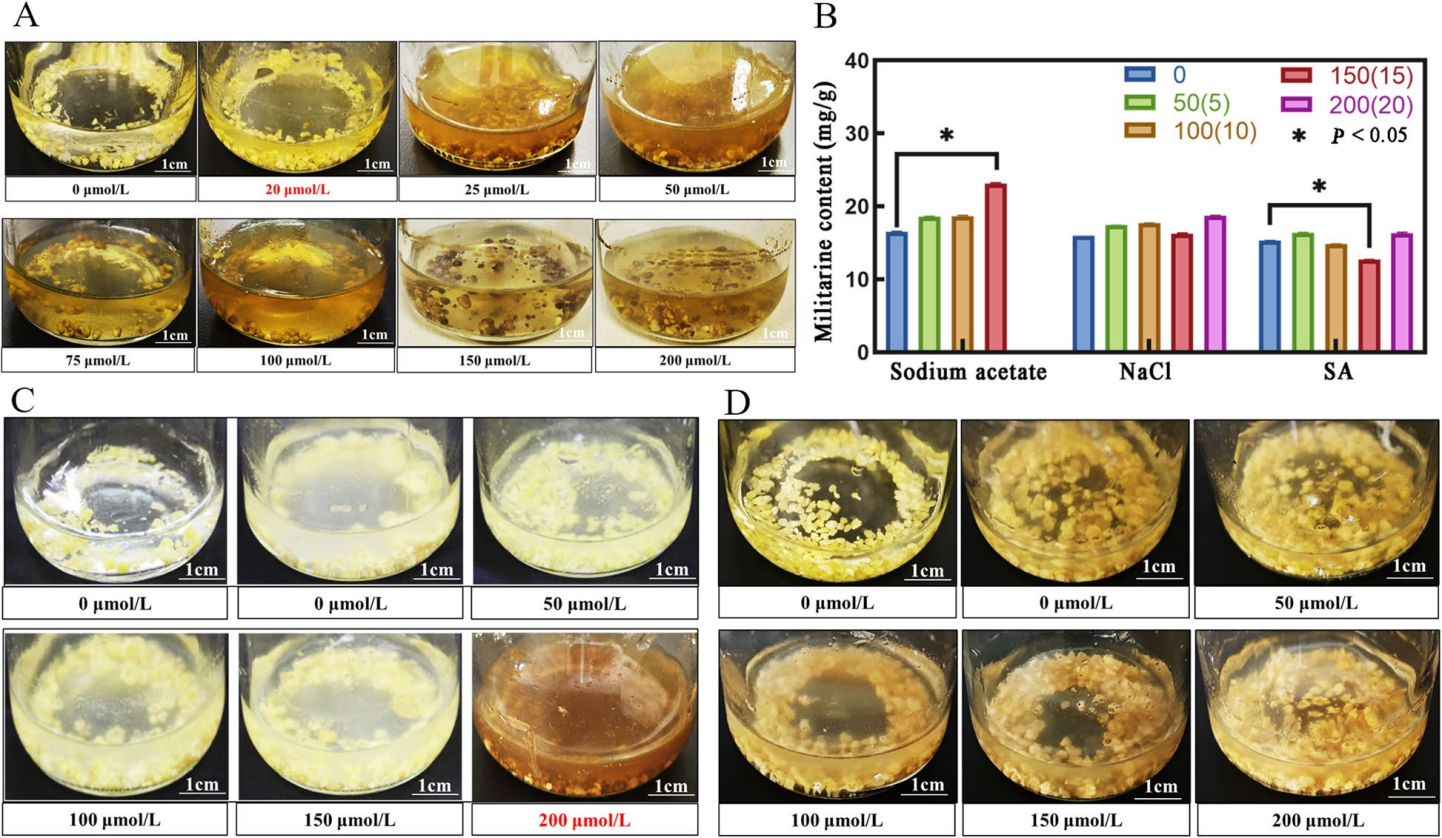
Growth status and content of militarine of *B. striata* in suspension calluses after elicitor culture. **A** Growth status of suspended calluses after SA culture, **B** Content of militarine in suspension calluses after elicitor culture, **C** Growth status of suspension calluses after sodium acetate culture. **D** The growth status of suspended calluses after NaCl culture

In order to ascertain the most effective concentration of elicitors for the production of militarine, suspension calluses were cultured using different concentrations of SA. The callus cultures that formed as a result exhibited different levels of necrosis, which were directly influenced by the dosage (Fig. 1A). When the suspension calluses were exposed to a concentration of 15 μmol/L SA within the range that allows for survival, there was a significant decrease (*P* < 0.05) in the production and buildup of militarine. The quantitative analysis showed that the militarine concentration was 12.68 mg/g, representing an 83% increase compared to the untreated control group (10.52 mg/g) (Fig. 1B). When NaAc was added for cultivation (as seen in Fig. 1C), the callus became brown and died at a concentration of 200 μmol/L. Nevertheless, when present at a concentration of 150 μmol/L, NaAc considerably increased (*P* < 0.05) the production and buildup of militarine. The concentration of militarine was 23.15 mg/g, representing a 1.4-fold increase compared to the control group. Across different NaCl concentrations (Fig. 1D), there were no notable variations in the levels of militarine (Fig. 1B), suggesting that the acetate ions were the active agents. The most effective concentration of culture for investigating the process of militarine production was found to be 150 μmol/L of NaAc or 15 μmol/L of SA when tested individually.

**Fig. 2:**
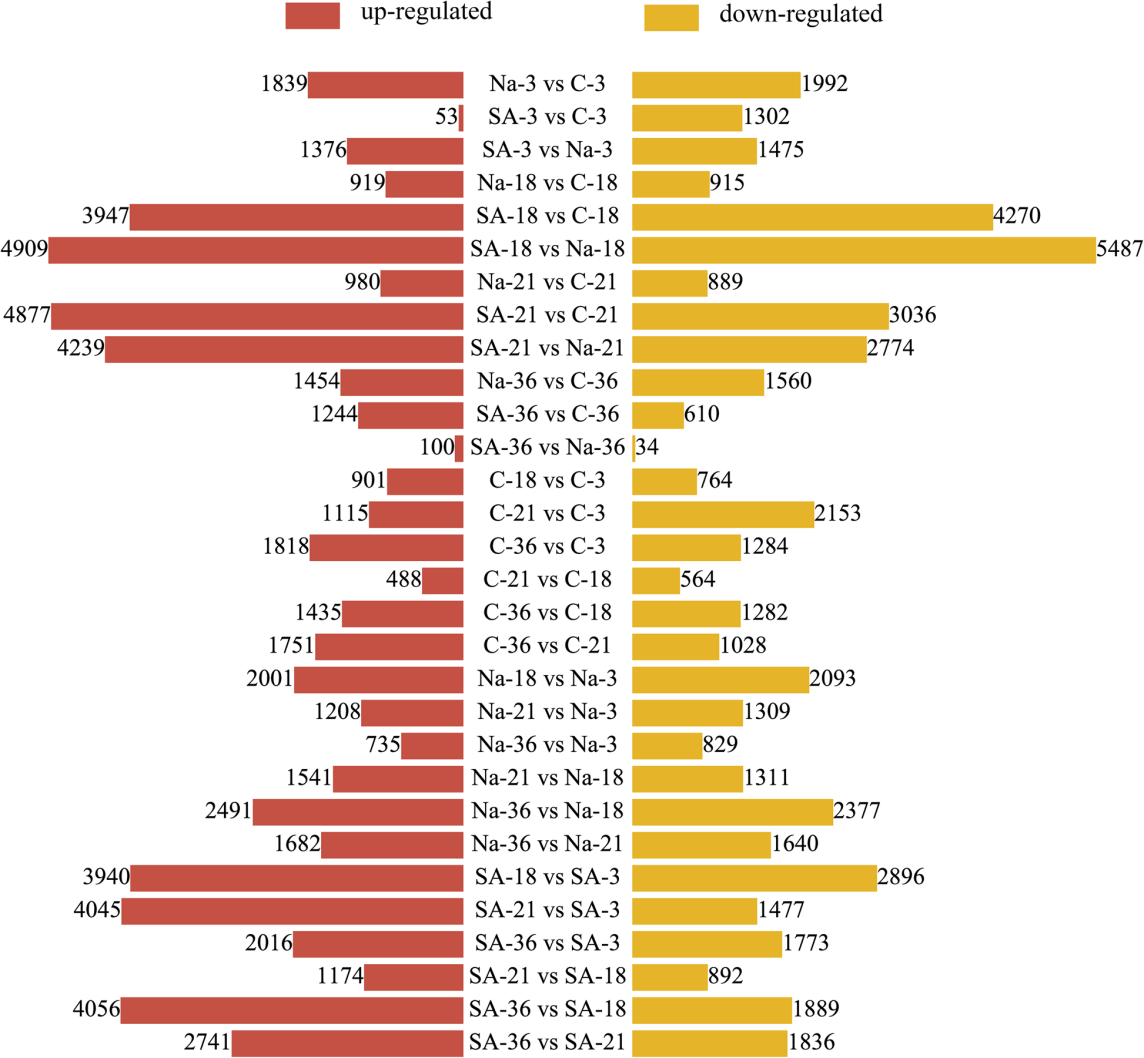
qPCR results. The ordinate corresponds to the average change of expression value, the abscissa represents different groups, and fpkm represents the value of transcriptome sequencing

Transcriptome sequencing was conducted on suspension-cultured calluses of *B. striata* at four distinct time points: 3 dpi, 18 dpi, 21 dpi, and 36 dpi. The sample approach and transcriptome method were described comprehensively in Sects. 2.1, 2.2, and 2.4. The process of sequencing produced a thorough collection of data, which was described in full in Table S3. The study categorised a total of 110,044 genes in six databases and conducted pairwise comparisons between them under various growth stage treatments and controls (Fig. 2). The study observed notable alterations in gene expression on the 18th and 21st days, leading to the identification of these time points as crucial for conducting functional pathway analysis. The research specifically examined the calluses and controls that were treated with SA (SA-18 vs C-18 and SA-21 vs C-21), as well as the nests and controls that were treated with NaAc (Na-18 vs C-18, Na-21 vs C-21). The examination of these comparisons unveiled distinct modifications in gene expression that could be the basis for the formation of secondary metabolites, such as militarine, triggered by the elicitor.

### qPCR results

**Fig. 3:**
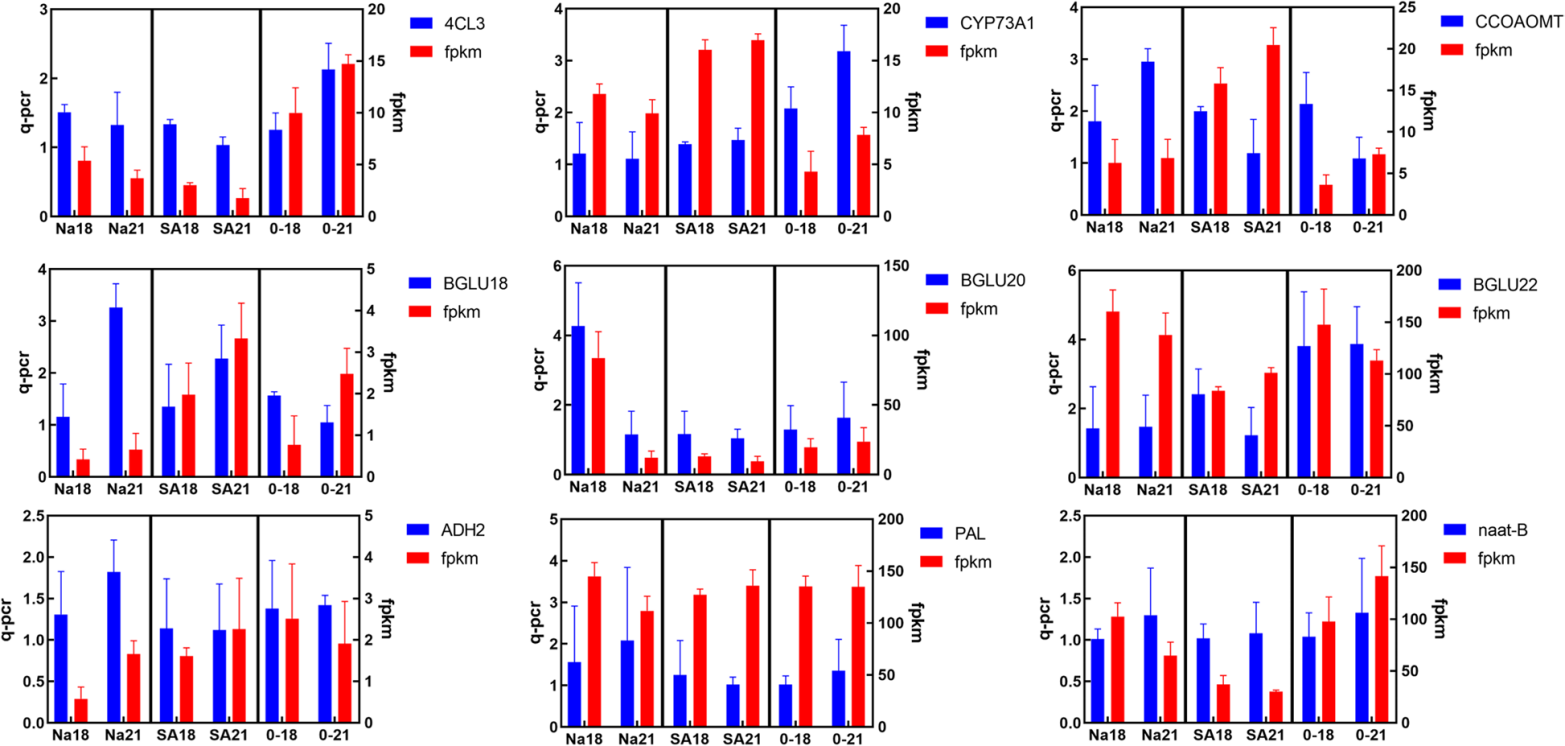
The number of upregulated and downregulated differentially expressed genes in different control groups. Red represents the number of upregulated genes in the comparison group, while yellow represents the number of downregulated genes in the comparison group. C represents blank control, Na represents sodium acetate, and SA represents salicylic acid. The second digit represents the number of days of cultivation, and the third digit represents repetition. Such as C_3 represents the first replicate on the third day of culture in the control group

To assess the precision of our transcriptome sequencing, we chose specific genes that play a role in the production pathway of militarine. We then developed primers using the gene sequence obtained from the transcriptome sequencing, which was relatively comprehensive. There was a total of 9 subgroups of DEGs that were analysed using qPCR. The reference gene used was glyceraldehyde 3-phosphate dehydrogenase (*GAPDH*). The qPCR results demonstrated that the expression patterns of these DEGs were consistent with the transcriptome data presented in Fig. 3. This finding serves as validation for the accuracy of our sequencing results and supports its utilisation in subsequent investigations.

### Transcriptomic analysis of militarine after elicitor culture

**Fig. 4:**
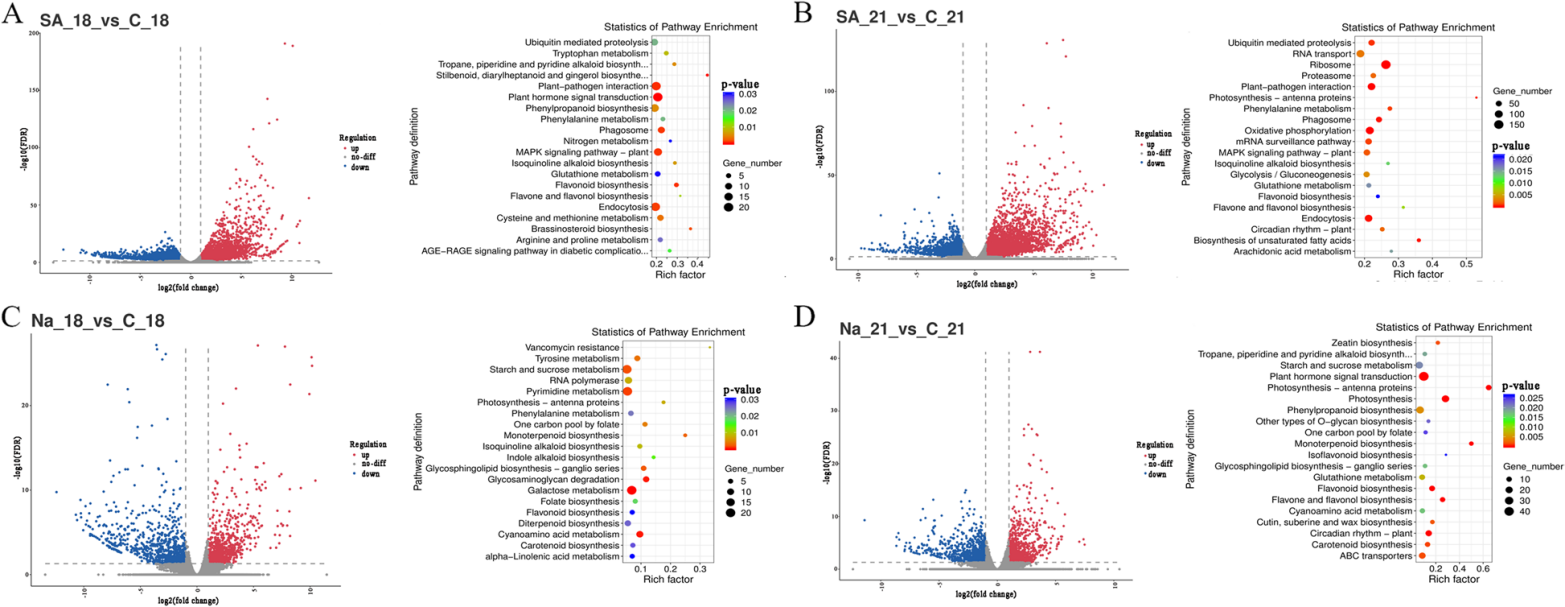
Volcano of gene expression in different comparison groups and enrichment of KEGG pathway in different comparison groups. A. SA-18 vs C-18, B. SA-21 vs C-21, C. Na-18 vs C-18, D. Na-21 vs C-21. C represents blank control, Na represents sodium acetate, and SA represents salicylic acid. The second digit represents the number of days of cultivation, and the third digit represents repetition. Such as C_18 represents the first replicate on the 18th day of culture in the control group

The findings of the transcriptional analysis (Fig. 4) showed that both SA and NaAc influenced the expression of a substantial number of genes in the suspension calluses of *B. striata*. Following NaAc treatment, the phenylpropanoid biosynthesis pathway (map00940) remained consistently enriched throughout time. This suggests that this pathway is essential for the production of militarine. The primary pathways of enrichment consisted of galactose metabolism (map00052), pyrimidine metabolism (map00240), aminose and nucleotide sugar metabolism (map00520), RNA transport (map03013), and phenylpropanoid biosynthesis (map00940), which suggested the production of substances necessary for the synthesis of militarine. Over time, there was a notable increase in the presence of plant hormone signal transduction (map04075), starch and sucrose metabolism (map00500), and interactions between plants and pathogens (map04626) in the pathways. This suggests a robust production of metabolites like glycosides, enabling cells to withstand stress. A total of 246 gene variants were identified in the differentially expressed genes, with 175 showed downregulation and 62 showing upregulation. The genes that showed significant expression were *BsGLB1*, *BsmalZ*, *BsUSP*, *BsUGP2*, *BsIAA*, *BsABF*, and *BsARRB*.

During the SA treatment, there was a notable enrichment of several key genes as time progressed. These genes include *BsSAUR50*, *BsSAUR32* (SAUR family protein, K14488), *BsWRKY 52* (WRKY transcription factor 52, K16225), and *BsCML* (calcium binding protein CML, K13448). Representative genes were *BsMRPL13* (large subunit ribosomal protein L13, K02871), *BsNDUFA12* (NADH quinone oxidoreductase subunit A, K11352), and *BsWRKY52*. Presented were enhanced pathways, including ribosome (map03010), oxidative phosphorylation (map00190), plant pathogen contact, endocytosis, and MAPK signalling pathway (Fig. 4). As the length of the elicitor stress increases, the number of genes that are expressed differently undergoes dynamic modifications.

The results suggest that the timing of processing has a considerable impact on gene expression in suspension callus tissues of *B. striata*, particularly in relation to the control of biosynthesis and metabolic pathways. Elicitors govern the growth, development, and nutritional metabolism of *B. striata* suspension callus tissue, establishing a foundation for the response mechanism to stress.

### Metabolomics analysis of militarine after elicitor culture

**Fig. 5:**
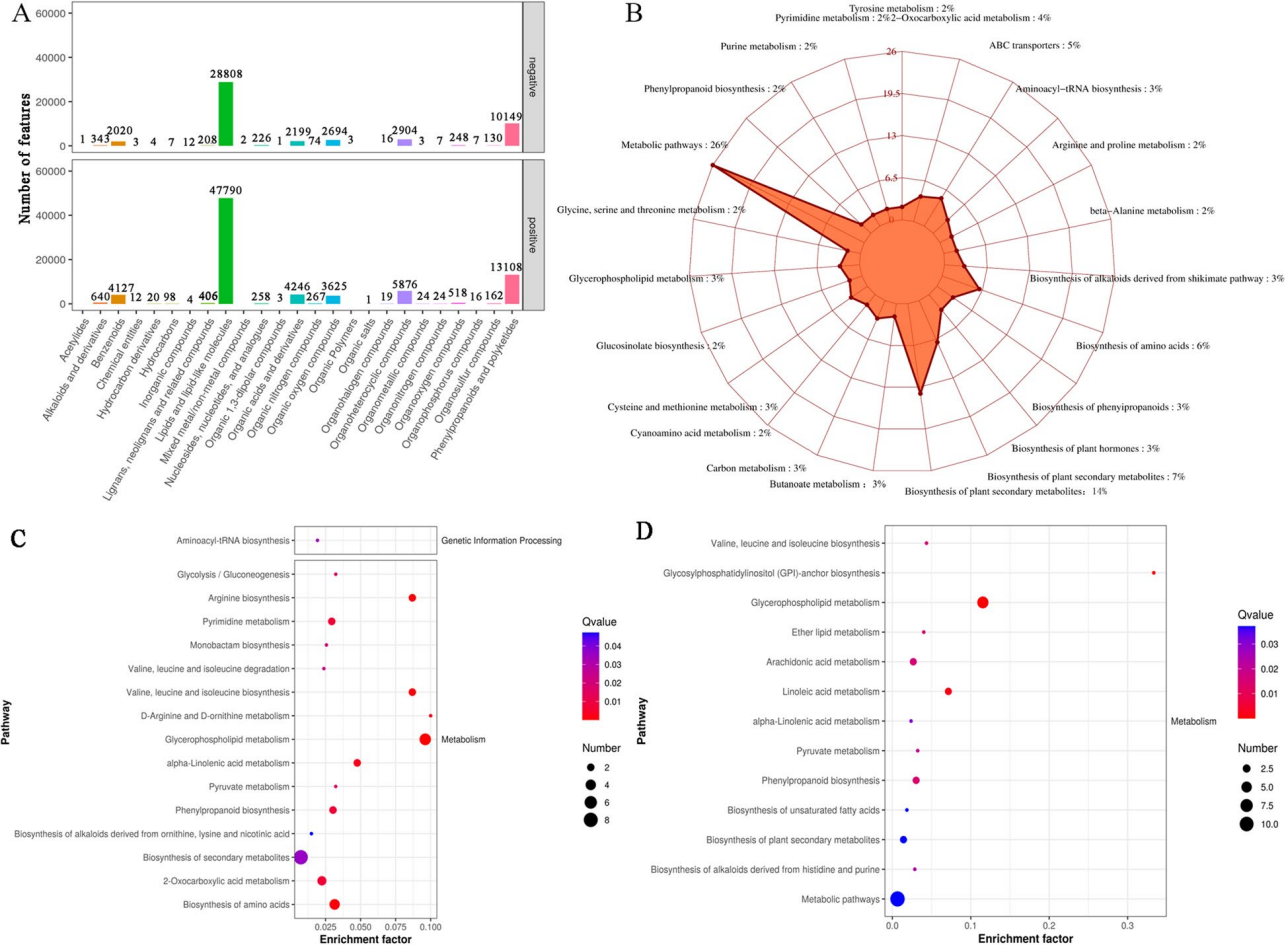
Classification and pathway enrichment of metabolites. **A** HMDB Super class classification diagram of metabolites. **B** Metabolite KEGG pathway enrichment radar chart. **C** Enrichment of differential metabolite pathway after sodium acetate treatment. **D** Enrichment of differential metabolite pathway after SA treatment

A total of 26,202 metabolites were discovered by metabolomics analysis. The categorization findings of the HMDB Super class (Fig. 5A) revealed that following elicitor treatment (Fig. 5C, D), a total of 3668 types of DAMs were identified, with 69 showing up-regulation and 66 showing down-regulation. The highest content among them was found in lipids and lipid molecules, followed by phenylpropane and polyketones, as well as organic acids and their derivatives, organic heterocyclic compounds, benzene ring compounds, etc. This included a total of 219 different components, such as quercetin, 4-coumarin, berberine, amino acids, L-arginine, phenylpyruvate, etc. These chemicals had the potential to synthesise phenolic acid molecules and their derivatives through a series of reactions. These processes were involved in the creation of militarine and may also contribute to peroxidation defence. The reaction had an impact on the biofilm system, subsequently influencing the movement of chemicals within the cell. The presence of flavonoids in it can halt the cascade of free radicals, therefore aiding in the suppression of the peroxidation reaction, which was the cellular response to stress. Another metabolite that showed a substantial enrichment was guanosine triphosphate (GMP), along with phosphatidylcholine (lecithin), phosphatidylcholine, phosphatidylethanolamine, and other metabolites. These metabolites were associated with the amplification of G protein linked signals in a cascade. The metabolic pathways were concentrated in amino acid and lipid metabolism pathways (Fig. 5B). The primary components of this process include metabolism, processing of environmental information, processing of genetic information, production of secondary metabolites, and transformation of chemical structures. The data suggested that the impact of elicitors on the production of militarine glycosides was primarily associated with the signal transduction of flavonoids, phenolic acids, and G protein coupling.

### Analysis of militarine biosynthetic pathways of DAMs and DEGs

To enhance comprehension of the connection between DEGs and DAMs involved in the production of militarine, scientists performed correlation analysis. They mapped these DEGs and DAMs to specific pathways in the KEGG pathway database, particularly the tyrosine metabolism pathway (map00350), phenylalanine metabolism pathway (map00360), and phenylpropanoid biosynthesis pathway (map00940).

**Fig. 6:**
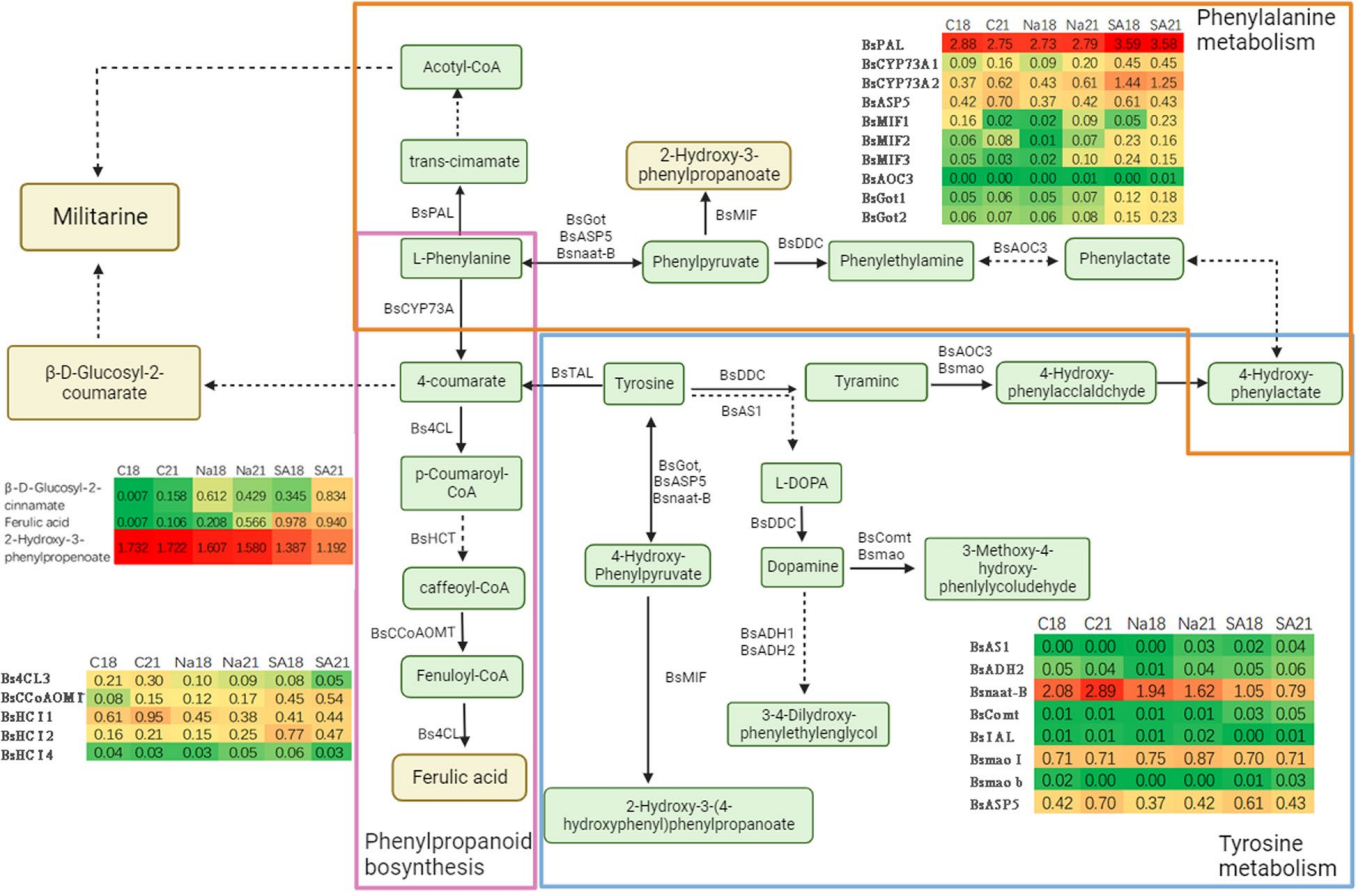
Network diagram and gene and metabolite expression heatmap of the synthesis process of militarine. According to the color code, each cell represents the normalized value of each gene and metabolite expression. Each value is the average of three biological replicates. *BsAOC3*, primary-amine oxidase; *BsADH1*, alcohol dehydrogenase; *BsDDC*, aromatic-L-amino-acid/L-tryptophan decarboxylase; *BsMIF*, phenylpyruvate tautomerase; *BsASP5*, toxoplasma Aspartyl Protease; *Bsnaat-B*, tyrosine aminotransferase; *Bs4CL*, 4-coumarate–CoA ligase; *BsGot*, aspartate aminotransferase; *BsComt*, catechol O-methyltransferase; *BsCYP73a*, trans-cinnamate 4-monooxygenase; *BsCCoAOMT*, caffeoyl-CoA O-methyltransferase; *BsPAL*, phenylalanine ammonia-lyase; *BsMao b/Mao I*, monoamine oxidase; *BsHCT*, hydroxycinnamoyl transferase

The heat map of gene expression (Fig. 6) revealed that following 18 days of NaAc treatment, the expression of *BsAOC3* (primary-amine oxidase, K00276), *BsADH1* (alcohol dehydrogenase, K13951), and *BsDDC* (aromatic-L-amino-acid/L-tryptophan decarboxylase, K01593) was increased compared to the control group. On the other hand, the expression of *BsMIF* (phenylpyruvate tautomerase, K07253) and *BsADH2* (K13951) was decreased. Following a 21-day NaAc treatment, the expression of *BsADH1* and *DDC* genes was increased, but the expression of *BsASP5* (Toxoplasma Aspartyl Protease, K00811), *Bsnaat-B* (tyrosine aminotransferase, TAT, K00815), *BsAOC3*, and *Bs4CL* (4-coumarate–CoA ligase, K01904) genes was decreased. Following 18 days of SA treatment, the genes *BsGot* (aspartate aminotransferase, cytoplasmic), *BsComt* (catechol O-methyltransferase, K00545), *BsCYP73a* (trans-cinnamate 4-monooxygenase, K00487), *BsCCoAOMT1* (caffeoyl-CoA O-methyltransferase, K00588), *BsPAL* (phenylalanine ammonia-lyase, K10775), and *BsMIF* exhibited increased expression, while *Bs4CL* and *BsAS1* showed decreased expression. Following a 21-day treatment with SA, the expression levels of *Bs4CL*, *BsASP5*, and *Bsnaat-B* were decreased, whereas the expression levels of *BsMaob* (monoamine oxidase, K00274), *BsMIF*, *BsComt*, *BsCYP73a*, *BsCCoAOMT1* (caffeoyl-CoA O-methyltransferase, K00588), *BsPAL*, and other genes were increased. Within the DAMs, there was a decrease in the accumulation of 2-hydroxy-3-phenylpropenoate (also known as phenylpropenoate ester or 2-Hydroxy-3-phenylpropenoate, with the chemical identifier C02763). On the other hand, there was an increase in the accumulation of ferulic acid (C01494) and cis-beta-D-Glucosyl-2-hydroxycinnamate (C05839).

### Network analysis and comprehensive mechanism model of militarine related DAM and DEG under elicito stress

**Fig. 7:**
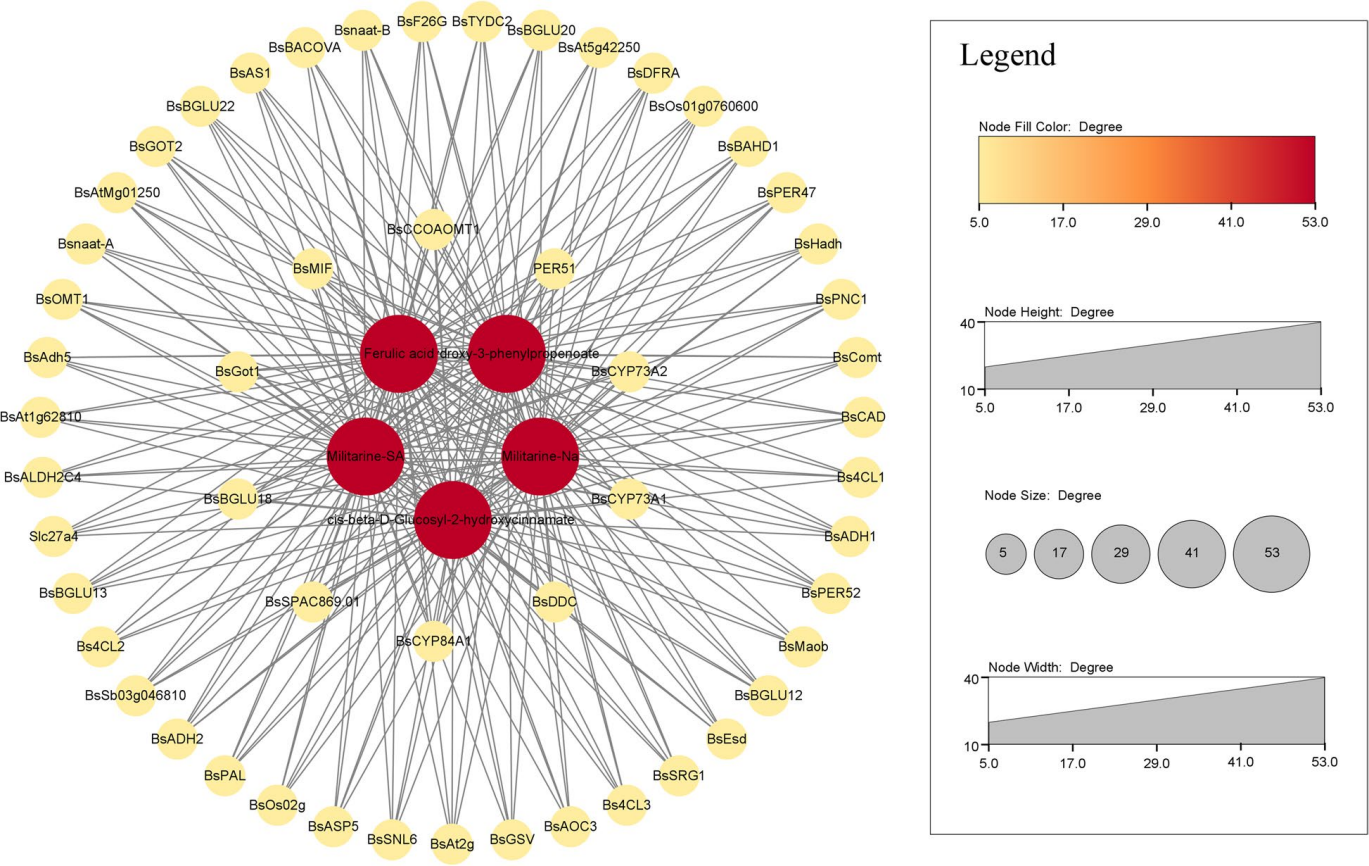
Gene and metabolite co-expression analysis based on Spearman correlation. Red represents metabolites, yellow represents genes. The thickness of the lines represents the strength of the association between metabolites and genes. The size of the circle usually indicates the abundance of metabolites or genes. The distance between circles indicates the relationship between metabolites or genes, while closer circles indicate closer relationships

Following the elimination of duplicate entries from the chosen genes and metabolites, a Spearman correlation analysis was performed (Fig. 7). *BsAOC3, BsBGLU20, BsBGLU22, BsComt, BsGOT2, BsADH1, BsMaob* were positively correlated with the content of militarine (*r* = 0.742, r = 0.742, *r* = 0.742, *r* = 0.72, *r* = 0.712, *r* = 0.713, *r* = 0.705), *BsGot1, BsCCOAOMT1, BsCYP73A1, BsCYP73A2, Bs4CL2, Bsnaat-B* were negatively correlated with the content of militarine (*r* = -0.765, *r* = -0.728, *r* = -0.713, *r* = -0.713, *r* = -0.713, *r* = -0.69, *r* = -0.69). *Bsnaat-B* were negative correlation with the content of ferulic acid (*r* = -0.69), *BsComt, BsADH1* were negative correlation with 2-Hydroxy-3-phenylpropenoate (*r* = -0.676, *r* = -0.669), using Cytocape to draw a co-expression network diagram of genes and metabolites.

**Fig. 8:**
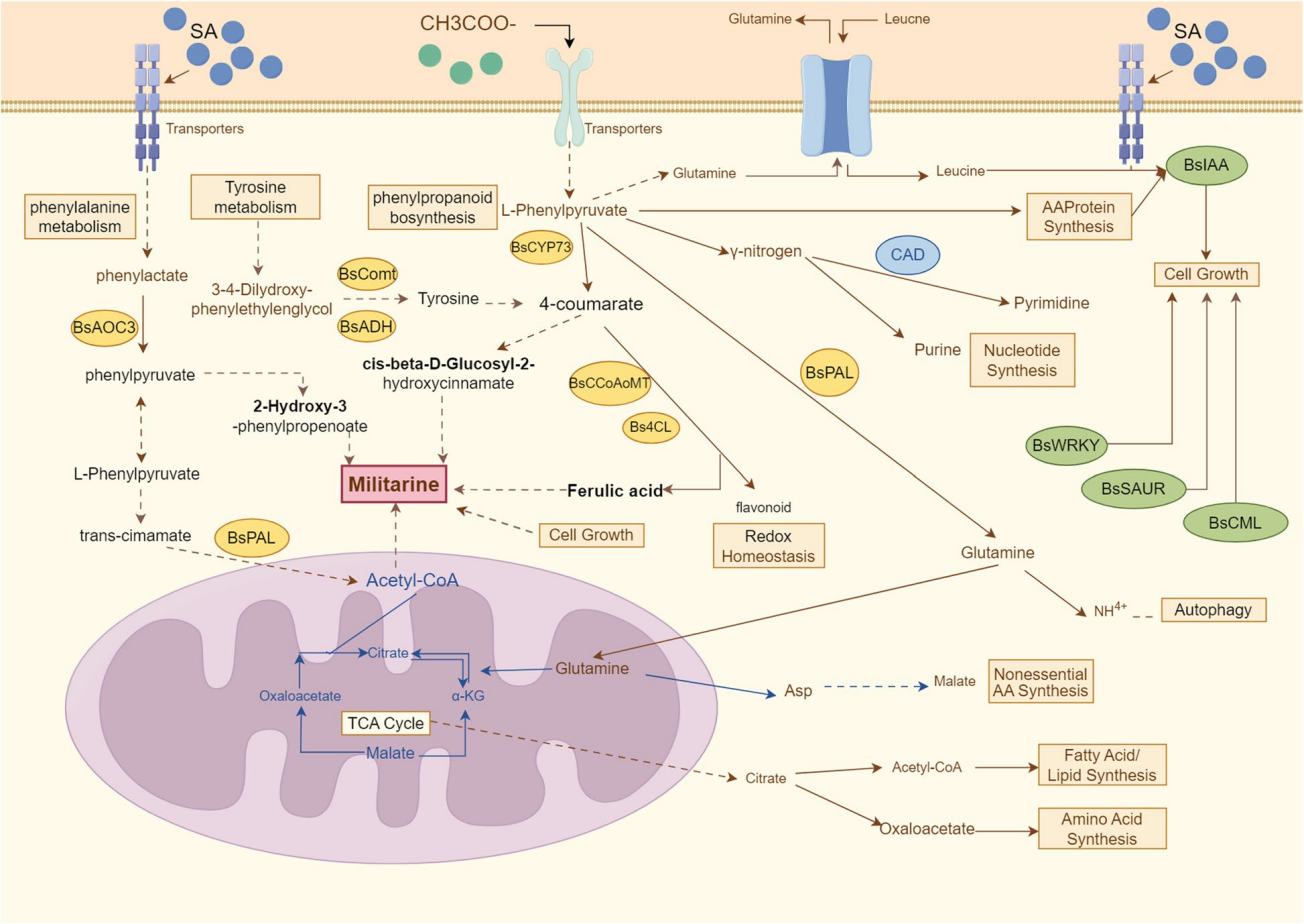
A model for the synthesis mechanism of militarine under elicitor stress. The yellow circle represents genes, the green circle represents gene families, the solid line represents direct involvement in the response, and the dashed line represents multiple steps in the middle

In the results provided above, suggest a model that explains the synthesis mechanism of militarine bases when subjected to elicitor stress. Illustrate the model using Figraw (Fig. 8). Following exposure to elicitor stress, suspension cells of *B.striata* activated defence mechanisms including as energy provision, amplification of signal transduction cascades, and control of antioxidant systems to manage the generated stress. Regarding energy provision, the buildup of amino acids like L-arginine and the vigorous metabolism of lipid molecules guarantee the functioning of proteins and biofilm systems. The primary components that accumulated in the antioxidant system were flavonoids, phenolic acids, and their precursors.

## Discussion

### The effect of elicitors on gene expression and metabolism

The objective of our work was to examine the impact of NaAc and SA on the gene expression and metabolic profiles of *B. striata* suspension calluses. The use of NaAc for elicitation led to the identification of 9,996 DEGs and 1,561 DAMs. On the other hand, the treatment with SA resulted in a more noticeable reaction, with 18,309 DEGs and 2,107 DAMs, suggesting a greater metabolic responsiveness to SA. The results of the NaAc therapy showed a notable increase in DEGs related to the metabolism of galactose. Notably, genes such as *BsGLB1* (Leclerc et al. 2023), *BsmalZ* (Wang et al. 2024), *BsUSP* (Althammer et al. 2022), and *BsUGP2* (Zeng et al. 2022), which were involved in the pentose phosphate pathway and gluconeogenesis, exhibited significant upregulation. This implied that NaAc may affect the production of sugar, which in turn affects the growth and metabolism of cells, aligning with the findings of Huang et al. (2022a, b).

SA treatment had a stronger impact on the signaling pathway of plant hormones, affecting cell growth by regulating the expression of gene families, and thereby regulating the synthesis of militarine. This was evident from the significant enrichment of DEGs such as *BsSAUR50* and *BsSAUR32* (Wang et al. 2022), *BsNDUFA12* (Li et al. 2023a), *BsCML* (Li et al. 2023b), *BsIAA3* (Wang, et al. 2023a), *BsABF* (Fiallos-Salguero et al. 2023), *BsARRB* (Yue et al. 2022), and *BsWRKY52* (Wang et al. 2023b). These genes were involved in multiple activities, such as translation initiation, oxidoreduction, plant defence signal transduction, growth, and stress response. In addition, the study by Zhang R et al. (2023) found that *BsADH1*, which plays a role in both glycolysis/gluconeogenesis and stress tolerance, showed a considerable enrichment. The heightened activity observed in the oxidative phosphorylation pathways indicated that the calluses treated with SA were well-equipped to handle metabolic alterations and facilitate the increased production and transportation of cellular substances. Both elicitors had an impact on important pathways such as plant hormone signalling, phenylpropanoid metabolism, and plant-pathogen interactions. Transcription factors and gene families associated with growth, development, and stress responses, such as *BsWRKY, BsIAA, BsSAUR*, and *BsCML*, showed a significant increase in abundance in response to both treatments. These findings suggest that NaAc and SA have an impact on both the growth and development of *B. striata* suspension calluses, as well as the activation of stress responses.

SA had been shown to be effective in enhancing the expression of these genes, while NaAc contributed to increase in the accumulation of precursor metabolites. The application of SA and NaAc as elicitors in *B. striata* suspension cultures showed promise in boosting the production of militarine. The study provides essential molecular insights that can be used as a foundation for the metabolic engineering of this medicinal chemical. These insights also open up possibilities for producing the compound on a larger scale and in a sustainable manner. Our research offers an innovative approach to enhance the production of valuable pharmacological chemicals in plant callus cultures, which will have a substantial impact on the pharmaceutical sector and the preservation of medicinal plants.

### The effect of elicitors on the biosynthesis of militarine

The biosynthesis of militarine, a secondary metabolite, was a complex process involving various substrates and enzymes (Li et al. 2020). Key substrates such as L-phenylalanine, L-tyrosine, and other phenolic precursors, undergo transformations through the shikimic acid and phenylpropanoid pathways (Feduraev et al. 2020; Fugate et al. 2023), catalyzed by enzymes like TAL (tyrosine ammonia lyase) (Huang YW et al., 2022), PAL (phenylalanine ammonia-lyase) (Gao et al. 2023), 4CL (4-coumarate–CoA ligase) (Ma et al. 2023) and CYP73a (Guo L et al., 2023). CoA played a crucial function in linking metabolic pathways, such as the breakdown of fatty acids and acetoacetic acid, and was essential for the generation of energy-rich molecules like NADPH/NADH during glycolysis and gluconeogenesis. Elicitors, whether they be biotic or abiotic factors, had a substantial influence on the production of militarine. They were recognised for their ability to regulate the amounts of gene expression related to amino acid metabolism and secondary metabolic pathways. For instance, elicitors can induce overexpression or suppression of genes encoding enzymes such as *BsTAL, BsPAL*, and *Bs4CL,* which were directly involved in the biosynthetic pathway of militarine. The stress response gene *TAT* (Ju X et al., 2023), the amino acid metabolism-related gene *BsGot* (Busch et al., 2021; Pezeshki et al. 2022), and the hormone metabolism-associated gene *BsComt* (Šanjug et al 2023) were also affected by elicitor treatment. Moreover, elicitors could influence the activity of *BsMIF* (Rosengren et al. 1997) and *BsAOC3* (Xu H et al., 2024) genes, which had roles in converting specific substrates in the metabolic pathways leading to militarine synthesis. The regulation of *BsCCoAOMT1* by elicitors affects the methylation process in the phenylpropanoid pathway, which could indirectly influence the production of militarine. In order to optimise the production of militarine, it was essential to have a thorough understanding of how elicitors affect the pathways and regulatory genes involved in its manufacture. This encompassed their influence on the shikimic acid pathway, the phenylpropanoid pathway, and the diverse metabolic links facilitated by CoA derivatives. Through the application of elicitors, it was feasible to manipulate the levels and activity of these enzymes, resulting in an augmentation of militarine production. This has the potential to greatly impact the commercial production and medicinal uses of militarine.

The administration of elicitors had a considerable impact on the phenolic acids, which act as intermediates in the production of militarine. The up-regulation of genes such as *Bs4CL, BsCCoAOMT, BsASP, BsAOC3* and *BsPAL* correlated with the altered accumulation of their products. The up-regulation specifically resulted in elevated concentrations of ferulic acid and cis-beta-D-Glucosyl-2-hydroxycinnamate. These compounds were found to be favourably correlated with the amount of militarine, indicating a synergistic influence on its production (Chen et al. 2020). In contrast, a rise in 2-Hydroxy-3-phenylpropenoate was associated with a decline in militarine. *BsAOC3* exhibited a positive link with militarine levels, but *Bsnaat-B* demonstrated negative relationships.

Ferulic acid, originating from the phenylpropanoid pathway, served as a crucial component of plant cell walls and possessed inherent antioxidant properties (Dong et al., 2022). The accumulation of this substance was a result of the cell's reaction to environmental stress and has the potential to impact the ageing and browning processes of cells. The process of browning, intensified by phenolic oxidation and tissue lignification, may have an adverse effect on the survival and development of cells (McLarin et al., 2020, Zhao et al. 2021).

In summary, elicitors had the ability to influence the production of militarine in suspension calluses of *B. striata* by impacting the creation of phenolic acid intermediates, such as ferulic acid. Acetate can regulate by influencing the acid–base environment in the organism, while SA can function as a transcription factor for regulation. This analysis of metabolic regulation provided prospective methods for enhancing the production of militarine by exploiting elicitor-mediated control.

## Conclusion

This study performed metabolomics and transcriptomics investigations to establish a putative mechanistic model for militarine. The addition of elicitors to the culture resulted in the observation that acetate ions and SA influence the synthesis of militarine. The regulation of this synthesis is mainly influenced by pathways such as phenylalanine metabolism, phenylpropanoid biosynthesis, and tyrosine metabolism. Several genes, including members of the *BsWRKY* transcription factor family, *BsIAA* gene family, *BsSAUR* gene family, *BsCML* gene family, play a role in regulating militarine synthesis. Metabolites such as ferulic acid, 2-Hydroxy-3-phenylpropenoate, and cis-beta-D-Glucosyl-2-hydroxycinnamate were involved in this process. Based on our findings, we hypothesized that the expression levels of *BsAOC3, BsComt, BsGOT2, BsADH1, BsPAL,* and *BsMaob* were positively correlated with the content of militarine, while the content of *BsGot1, BsCCOAOMT1*, *BsCYP73a1, BsCYP73a2, Bs4CL2,* and *Bsnaat-B* were negatively correlated with militarine content. These findings enhance our comprehension of how militarine biosynthesis responds to elicitors, establishing a basis for widespread industrial manufacturing and possible medical uses.

## Supplementary Information

Supplementary Material 1: Table S1 HPLC gradient elution program.

Supplementary Material 2: Table S2 The information of qPCR primers.

Supplementary Material 3: Table S3 Summary of quality of raw sequencing data.

## Data Availability

The data presented in the study are deposited in the NCBI repository, accession number PRJNA1009214.
