# Supplementary material for: Integrated transcriptomics and metabolomics provide insights into the biosynthesis of militarine in the cell suspension culture system of Bletilla striata
Source: Adv Biotechnol (Singap). 2024 Jul 16;2(3):25. doi: 10.1007/s44307-024-00032-w (PMC11740853; doi:10.1007/s44307-024-00032-w)
Supplement: Supplementary file 1 — Supplementary Material 1: Table S1 HPLC gradient elution program. [file 44307_2024_32_MOESM1_ESM.docx]

**Table S1**

| Time(min) | Acetonitrile (100%) | 0.1% Phosphoric acid (%) |
| --- | --- | --- |
| 0 | 20 | 80 |
| 10 | 20 | 80 |
| 25 | 50 | 50 |
| 27 | 95 | 5 |
| 30 | 95 | 5 |
| 32 | 100 | 0 |
| 45 | 100 | 0 |
