# Supplementary material for: Integrated transcriptomics and metabolomics provide insights into the biosynthesis of militarine in the cell suspension culture system of Bletilla striata
Source: Adv Biotechnol (Singap). 2024 Jul 16;2(3):25. doi: 10.1007/s44307-024-00032-w (PMC11740853; doi:10.1007/s44307-024-00032-w)
Supplement: Supplementary file 2 — Supplementary Material 2: Table S2 The information of qPCR primers. [file 44307_2024_32_MOESM2_ESM.docx]

**Table S2**

| Gene | | Sequence (5'->3') | Tm | GC% |
| --- | --- | --- | --- | --- |
| Bs4CL3-F | CACTCCCGCAGAAATCCACA | | 60.32 | 55 |
| Bs4CL3-R | CGGAATTTCGGCCTCGTTTG | | 60.18 | 55 |
| BsADH2-F | CAAGCCTCGTTCTGACATACCT | | 60.09 | 50 |
| BsADH2-R | GTTTTTAGAACTGCCCCCAACT | | 59.04 | 45.45 |
| BsBGLU18-F | AGGAGGCTCTATTGGCATTGT | | 59.15 | 47.62 |
| BsBGLU18-R | GCAGCAGCCATTCGTTCTAC | | 59.62 | 55 |
| BsBGLU20-F | GGATGTGGCATCTGACGGATA | | 59.66 | 52.38 |
| BsBGLU20-R | GATAGCTCCTCGTCCATCAGG | | 59.46 | 57.14 |
| BsBGLU22-F | TCTTCCAAGGCAGGCATTCA | | 59.59 | 50 |
| BsBGLU22-R | AAACGGGCTATGGGATGGTG | | 60.11 | 55 |
| BsCCOAOMT1-F | TCTGCCGTCGGATTAGCTG | | 59.56 | 57.89 |
| BsCCOAOMT1-R | GCAAATGCAGACACAACGCC | | 61.28 | 55 |
| BsCYP73A1-F | ACTCGGTCCCATAAGCCAGA | | 60.33 | 55 |
| BsCYP73A1-R | TCCAGCGGCCATTCTTTTCT | | 59.96 | 50 |
| BsPAL-F | TTGAAAGAAAATGGAGAACGGGC | | 59.99 | 43.48 |
| BsPAL-R | GTCCCTCCATTACTGCTACCC | | 59.58 | 57.14 |
| Bsnaat-B-F | GGCTACTGTCACCTCCACAC | | 60.04 | 60 |
| Bsnaat-B-R | TCTCATGGAGATGGACGGGT | | 60.03 | 55 |
| GAPDH-F | GCATCCTGGGCTACACTGAG | | 60.18 | 60 |
| GAPDH-R | GTCAAAGGTGGAGGAGTGGG | | 59.96 | 60 |
