# Supplementary material for: Integrated transcriptomics and metabolomics provide insights into the biosynthesis of militarine in the cell suspension culture system of Bletilla striata
Source: Adv Biotechnol (Singap). 2024 Jul 16;2(3):25. doi: 10.1007/s44307-024-00032-w (PMC11740853; doi:10.1007/s44307-024-00032-w)
Supplement: Supplementary file 3 — Supplementary Material 3: Table S3 Summary of quality of raw sequencing data. [file 44307_2024_32_MOESM3_ESM.docx]

**Table S3**

| Sample | Raw_Reads | Raw_Bases | Valid_Reads | Valid_Bases | Valid% | Q20% | Q30% | GC% |
| --- | --- | --- | --- | --- | --- | --- | --- | --- |
| C_18_1 | 40173838 | 6.03G | 39718226 | 5.89G | 98.87 | 98.04 | 93.42 | 45.01 |
| C_18_2 | 39777276 | 5.97G | 39338326 | 5.84G | 98.90 | 98.05 | 93.47 | 44.91 |
| C_18_3 | 37506496 | 5.63G | 37089392 | 5.51G | 98.89 | 98.13 | 93.66 | 44.71 |
| C_21_1 | 39003576 | 5.85G | 38564996 | 5.72G | 98.88 | 98.03 | 93.41 | 44.61 |
| C_21_2 | 39151682 | 5.87G | 38735188 | 5.75G | 98.94 | 98.11 | 93.61 | 44.94 |
| C_21_3 | 41865594 | 6.28G | 41407990 | 6.15G | 98.91 | 98.11 | 93.62 | 45.00 |
| C_3_1 | 41108916 | 6.17G | 40609006 | 6.02G | 98.78 | 97.80 | 92.85 | 45.20 |
| C_3_2 | 41458320 | 6.22G | 40938712 | 6.07G | 98.75 | 97.81 | 92.91 | 45.24 |
| C_3_3 | 43168988 | 6.48G | 42624266 | 6.32G | 98.74 | 97.78 | 92.81 | 45.32 |
| C_36_1 | 51977946 | 7.80G | 51282238 | 7.60G | 98.66 | 97.95 | 93.24 | 45.58 |
| C_36_2 | 39042824 | 5.86G | 38605048 | 5.73G | 98.88 | 98.17 | 93.78 | 44.59 |
| C_36_3 | 52899166 | 7.93G | 52208464 | 7.74G | 98.69 | 97.95 | 93.23 | 45.43 |
| Na_18_1 | 39301618 | 5.90G | 38844754 | 5.76G | 98.84 | 97.85 | 92.95 | 44.92 |
| Na_18_2 | 39037362 | 5.86G | 38580338 | 5.72G | 98.83 | 97.84 | 92.96 | 44.87 |
| Na_18_3 | 39977090 | 6.00G | 39479646 | 5.85G | 98.76 | 97.89 | 93.06 | 44.89 |
| Na_21_1 | 41521652 | 6.23G | 41043616 | 6.09G | 98.85 | 97.90 | 93.08 | 44.85 |
| Na_21_2 | 41784928 | 6.27G | 41298218 | 6.12G | 98.84 | 97.84 | 92.96 | 45.00 |
| Na_21_3 | 43134406 | 6.47G | 42628890 | 6.32G | 98.83 | 97.88 | 93.02 | 44.92 |
| Na_3_1 | 41342300 | 6.20G | 40478196 | 6.00G | 97.91 | 98.04 | 93.43 | 44.74 |
| Na_3_2 | 40352972 | 6.05G | 39702674 | 5.88G | 98.39 | 97.92 | 93.13 | 44.96 |
| Na_3_3 | 37506812 | 5.63G | 37015132 | 5.49G | 98.69 | 97.92 | 93.13 | 45.13 |
| Na_36_1 | 39499428 | 5.92G | 39027020 | 5.78G | 98.80 | 97.90 | 93.09 | 44.59 |
| Na_36_2 | 37442760 | 5.62G | 37017596 | 5.49G | 98.86 | 97.84 | 92.94 | 44.85 |
| Na_36_3 | 40087414 | 6.01G | 39606250 | 5.87G | 98.80 | 97.93 | 93.16 | 44.02 |
| SA_18_1 | 53073158 | 7.96G | 52369766 | 7.76G | 98.67 | 97.91 | 93.17 | 45.32 |
| SA_18_2 | 53633140 | 8.04G | 52913988 | 7.84G | 98.66 | 97.98 | 93.33 | 45.38 |
| SA_18_3 | 50077672 | 7.51G | 49406960 | 7.32G | 98.66 | 97.98 | 93.32 | 45.53 |
| SA_21_1 | 54614890 | 8.19G | 53854654 | 7.98G | 98.61 | 97.91 | 93.18 | 45.24 |
| SA_21_2 | 54895018 | 8.23G | 54174408 | 8.03G | 98.69 | 97.92 | 93.16 | 45.36 |
| SA_21_3 | 41427198 | 6.21G | 40856852 | 6.04G | 98.62 | 97.61 | 92.44 | 45.39 |
| SA_3_1 | 39550446 | 5.93G | 39099768 | 5.80G | 98.86 | 97.91 | 93.13 | 44.95 |
| SA_3_2 | 40133062 | 6.02G | 39657526 | 5.88G | 98.82 | 97.93 | 93.17 | 45.19 |
| SA_3_3 | 38931156 | 5.84G | 38456742 | 5.70G | 98.78 | 98.03 | 93.42 | 44.81 |
| SA_36_1 | 41819244 | 6.27G | 41301028 | 6.12G | 98.76 | 97.85 | 92.98 | 44.78 |
| SA_36_2 | 41260868 | 6.19G | 40580476 | 6.01G | 98.35 | 97.83 | 92.91 | 44.79 |
| SA_36_3 | 54867954 | 8.23G | 54172214 | 8.02G | 98.73 | 97.80 | 92.88 | 44.74 |

Annotation: C represents blank control, Na represents sodium acetate, and SA represents salicylic acid. The second digit represents the number of days of cultivation, and the third digit represents repetition. Such as C_18_1 represents the first replicate on the 18th day of culture in the control group. Mature capsules were induced in the induction medium and cultivated for 30 days. Post induction, soft and tender yellow tissues were selected for inoculation into the subculture medium, with the medium refreshed every 15 days. Following the second subculture (recorded as 0 days post-inoculation, dpi), at different growth stages (3, 18, 21, 36 dpi), suspension cell samples were randomly selected based on random sampling principles, with three biological replicates for each time point.
